# The First Electrochemical MIP Sensor for Tamoxifen

**DOI:** 10.3390/s140507647

**Published:** 2014-04-25

**Authors:** Aysu Yarman, Frieder W. Scheller

**Affiliations:** 1 Fraunhofer Institute for Biomedical Engineering IBMT, Am Mühlenberg 13, 14476 Potsdam, Germany; E-Mail: yarman@uni-potsdam.de; 2 Institute of Biochemistry and Biology, University of Potsdam, Karl-Liebknecht-Str. 24-25, 14476 Potsdam, Germany

**Keywords:** molecularly imprinted polymers, anticancer drug, tamoxifen, electropolymerisation

## Abstract

We present an electrochemical MIP sensor for tamoxifen (TAM)—a nonsteroidal anti-estrogen—which is based on the electropolymerisation of an *O*-phenylenediamine–resorcinol mixture directly on the electrode surface in the presence of the template molecule. Up to now only “bulk” MIPs for TAM have been described in literature, which are applied for separation in chromatography columns. Electro-polymerisation of the monomers in the presence of TAM generated a film which completely suppressed the reduction of ferricyanide. Removal of the template gave a markedly increased ferricyanide signal, which was again suppressed after rebinding as expected for filling of the cavities by target binding. The decrease of the ferricyanide peak of the MIP electrode depended linearly on the TAM concentration between 1 and 100 nM. The TAM-imprinted electrode showed a 2.3 times higher recognition of the template molecule itself as compared to its metabolite 4-hydroxytamoxifen and no cross-reactivity with the anticancer drug doxorubucin was found. Measurements at +1.1 V caused a fouling of the electrode surface, whilst pretreatment of TAM with peroxide in presence of HRP generated an oxidation product which was reducible at 0 mV, thus circumventing the polymer formation and electrochemical interferences.

## 1. Introduction

During natural evolution highly competent biocatalysts and binders have evolved from very simple components. Molecular recognition takes place in so-called binding sites, e.g., the paratope of antibodies, which typically comprise 10–15 amino acids. In order to mimic the binding by antibodies and the catalytic activity of enzymes fully synthetic functional polymers have been created by co-polymerising a functional monomer and a cross-linker in the presence of the target analyte. In the pre-polymerisation mixture, the dissolved target interacts by covalent (pre-organised approach) or non-covalent (self-assembly approach) binding with the functional monomer and in the subsequent polymerisation the shape of the target molecule is “imprinted” by the reaction with the cross-linker. After polymerisation the template molecules are removed, providing binding sites ideally complementary in size, shape and functionality to the template, thus the template preferentially rebinds to the cavity. Bulk polymerisation is most frequently used for the preparation of molecularly imprinted polymers (MIPs). Their synthesis and application frequently requires the presence of non-aqueous solvents and they frequently show slow target binding due to the restricted transport in the bulk phase.

A large spectrum of MIPs has been developed for the application in chromatography and sensors [1–4]. However, the affinity and especially the selectivity of MIPs are in general lower than those of their biological counterparts. Furthermore, for analytical applications of MIPs the generation of the measuring signal is still a challenge. Thus, combination of MIPs with enzymes should improve the analytical performance of sensors. In fact, enzyme–labelled “tracers” have been used in analogy to competitive immunoassays also in MIP sensors, e.g., for oxytetracycline [5]. On the other hand, the harsh conditions of MIP preparation have restricted the integration of enzymes. Very recently we presented a surface architecture which comprises a substrate-converting enzyme layer on top of a product-imprinted electrode [6]. For the analgesic drug aminopyrine this combination resulted in the elimination of interferences by ascorbic acid and uric acid.

**Figure 1.**
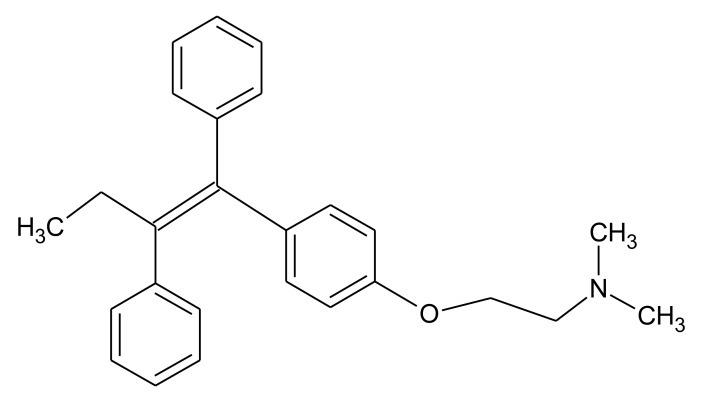
Structure of tamoxifen.

In this paper we present preliminary results of an electrochemical MIP sensor for tamoxifen—a nonsteroidal anti-estrogen which is used in the therapy of invasive human breast cancer (Figure 1). It has been banned by the International Olympic Committee and the indication of metabolites in urine is considered a proof of doping. This study is based on the electropolymerisation of *O*-phenylene-diamine-resorcinol mixture directly on the electrode surface in the presence of the template molecule tamoxifen. Furthermore, a concept is discussed for the combination of the respective MIP with the enzymatic conversion of the drug in order to decrease the influence of interfering substances.

## 2. Experimental Section

### 2.1. Chemicals

*O*-Phenylenediamine dihydrochloride (*O*-PD), resorcinol (Res), 4-hydroxytamoxifen and doxorubicin hydrochloride were purchased from Sigma-Aldrich (Steinheim, Germany) and tamoxifen from Molekula (München, Germany). All reagents were of analytical grade and used without further purification.

### 2.2. Preparation of Electrodes

Glassy carbon disk electrodes (GCE) (3 mm in diameter) were used for the voltammetric and amperometric measurements. Prior to electropolymerisation, electrodes were cleaned with ethanol and treated with 60% nitric acid for 15 min. After this, mechanical cleaning was performed with 1.0, 0.3 and 0.05 μm alumina slurry, respectively and electrodes were rinsed with Millipore water (Type 1) by sonication.

TAM-imprinted GCEs were prepared in 5 mM *O*-PD:5 mM resorcinol mixture (20% methanol containing 80 mM acetate buffer, pH 5.2) containing 0.4 mM TAM by cyclic voltammetry sweeping between 0 and 0.8 V (20 scans) at a scan rate of 50 mV/s. Non-imprinted electrodes were prepared in a similar way in the absence of template. Template molecules were removed by the treatment with the mixture of methanol-water-1 M NaOH (2:1:1, *v/v/v*) at 60 °C for 1 h shaking with a speed of 300 rpm.

### 2.3. Apparatus and Electrochemical Measurements

Electrochemical measurements were performed in a stirred electrochemical cell with a three-electrode configuration using a PalmSens potentiostat (Utrecht, The Netherlands). A glassy carbon disk electrode (GCE) with a diameter of 3 mm was used as the working electrode, an Ag/AgCl (in 3 M KCl solution) electrode was the reference electrode, and a platinum wire served as the counter electrode.

TAM rebinding studies were performed in 10 mM ferricyanide solution (in 100 mM KCl) sweeping between −0.2 and 0.8 V (three scans) at a scan rate of 50 mV/s.

Amperometric measurements were performed under aerobic conditions in 85 mM acetate buffer containing 15% methanol (*v/v*) at pH 5.2. A working potential of +1.1 V was applied. After baseline stabilisation had occurred, the current was recorded after TAM addition (2 mM stock in methanol) into the reaction chamber as a function of time. All the experiments were carried out at room temperature.

## 3. Results and Discussion

### 3.1. Generation of the MIPs and Characterisation with a Redox Marker

**Figure 2.**
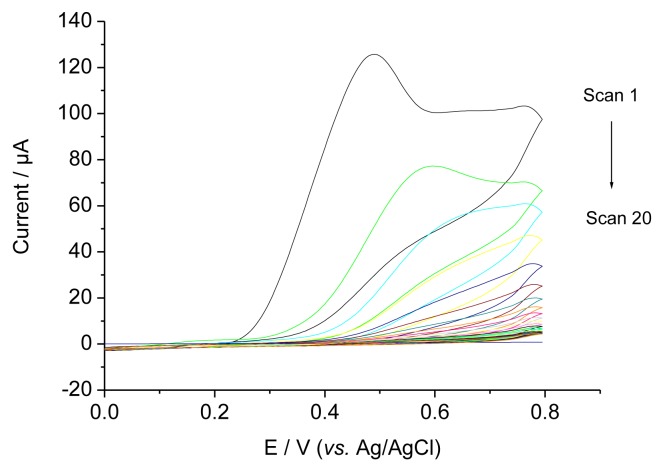
CVs showing formation of TAM-MIP.

Figure 2 shows CVs during the electropolymerisation (EP) of a *O*-PD-Res mixture on a glassy carbon electrode in the presence of 0.4 mM TAM. In the first scan an irreversible peak was obtained between 400 and 450 mV. The current decreased with the subsequent sweeps and approached zero, indicating the formation of a non-conducting film on the electrode surface [7]. Because TAM is not electroactive in the potential range, similar CVs were obtained in the presence and absence of TAM.

**Figure 3.**
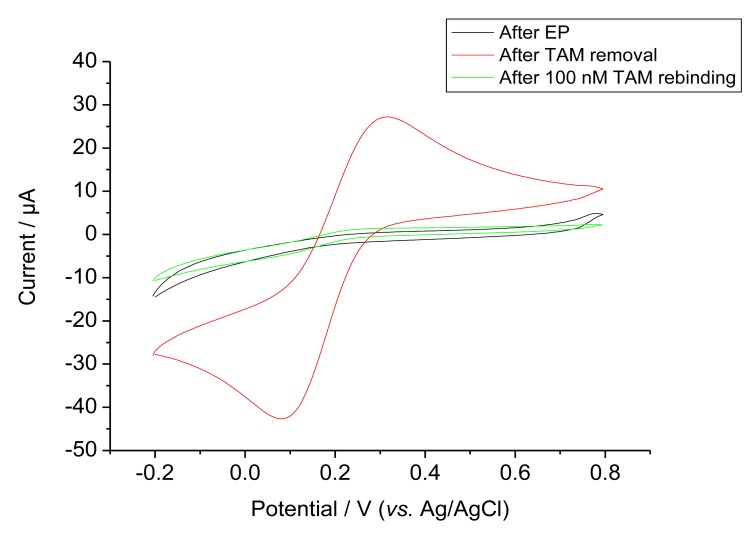
Overlay of CVs of MIP electrode after electropolymerisation (black), after TAM removal (red), and after TAM rebinding (green) in 10 mM ferricyanide at a scan rate of 50 mV/s.

Ferricyanide was used as a redox probe in order to characterise the permeability after EP, template removal and rebinding, Figure 3 shows the cyclic voltammograms of these steps. Bare GCE gave the highest response (not shown). On the other hand, after EP the current for ferricyanide was almost completely suppressed for both the MIP and control NIP. The MIP modified electrode gave a markedly increased ferricyanide signal after the removal of the template by incubation in the alkaline solution. This signal was again suppressed after rebinding as expected for filling cavities by target binding. This rebinding of the target was completed after 1 h.

**Figure 4.**
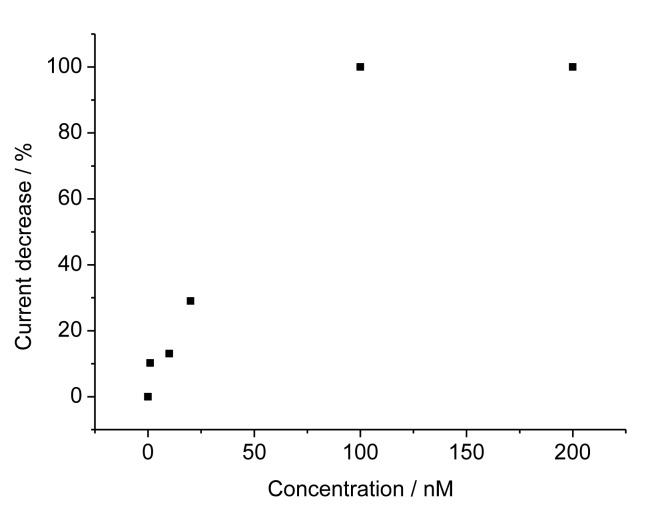
Concentration dependence for tamoxifen at TAM-MIP.

For the TAM-imprinted MIP the peak currents for the redox marker ferricyanide decreased with increasing concentration of TAM. The relative current decrease depends linearly on the TAM concentration from 1 to 100 nM and it reaches saturation above that level (Figure 4). These values show that our surface–imprinted MIP has fast rebinding and a measuring range at more than 100-fold lower concentrations than the bulk MIPs described in literature [8–11]. The TAM concentration in serum after the intake of the typical doses in breast cancer treatment of 20 mg is in the range between 50 and 300 nM. Thus our MIP sensor covers the relevant concentration range after a 1:10 dilution of the serum samples.

For the non-imprinted polymer the addition of TAM has a negligible effect on the peaks for ferricyanide. Thus a calculation of an “imprinting factor” is meaningless.

Additionally, cross-reactivity studies were performed. Interestingly, no cross-reactivity with doxorubicin, another anticancer drug, was found. Furthermore, the signal for binding of 4-hydroxytamoxifen, which is an intermediate in the hepatic metabolism of tamoxifen, is almost 2.3 times smaller than for the target at the TAM-imprinted electrode. This shows that the TAM imprinted electrode preferentially recognises the template molecule itself.

In the literature there are only a few papers describing MIPs for tamoxifen and its metabolites. All MIPs are bulk polymers based on methacrylic acid derivatives as functional monomers. These interact with the ternary amine function of the target. Copolymerisation with styrene resulted in an enhanced affinity by the π-π interaction with the aromatic rings of tamoxifen [11]. Acetonitrile (ACN) was used as porogen and ACN/acetic acid/water mixtures for the removal of the hydrophobic template. The grounded bulk polymers were packed in chromatography columns and applied for solid phase extraction before HPLC-UV analysis of tamoxifen containing urine samples [11].The imprinting factor (for 4-hydroxytamoxifen), *i.e.*, the ratio of target binding to MIP and the non-imprinted control increased from 0.6 for pure acetonitrile up to 7.1 in a ACN/acetic acid mixture. Interestingly, a propranolol–imprinted polymer showed stronger binding for tamoxifen than the MIP using TAM as the template [8,9]. Application of formaldehyde–amplified chemiluminescence of the Mn(IV) catalysed oxidation of tamoxifen in a MIP column brought about a measuring range between 0.1 and 6 mg/L [10].

### 3.2. Anodic Oxidation of TAM at the MIP Covered Electrode

**Figure 5.**
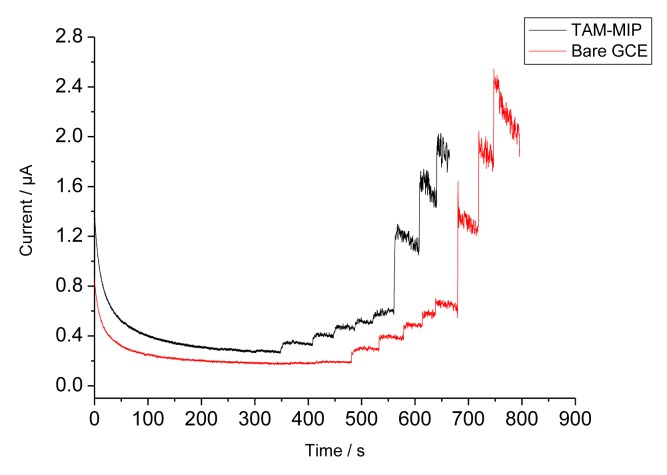
Amperometric responses of bare GCE (red) and TAM-MIP (black) on stepwise addition of 5 × 1 and 3 × 10 μM TAM on different electrodes in 85 mM acetate buffer containing 15% methanol at pH 5.2.

Since TAM generates an oxidation current above 900 mV [12–14], the binding of TAM to the MIP could also be investigated by measuring the anodic current at +1,100 mV. The amperometric responses of the bare GCE and the MIP covered electrode during stepwise addition of TAM are shown in Figure 5. Whilst at the NIP only a negligible current increase is found, the appearance of currents indicates that TAM can permeate through the pores of the electropolymer. However, at higher concentrations the anodic oxidation of TAM resulted in a polymer film which caused fouling of the electrode surface as reflected by the decreased current. The current of the MIP electrode increases linearly from 1 to 15 μM and it amounts to almost 80% of that of the bare electrode. However, the signal generation by the anodic oxidation of TAM leads to a signal decrease in the subsequent measurement, obviously due to the formation of an insulating film. Therefore this measuring principle is not appropriate for the characterisation of the MIP.

In order to prevent the fouling of the electrode surface, a lower potential has to be applied. Recently Radhapyari *et al.* reported that adsorption of horseradish peroxidase (HRP) to a polyaniline covered electrode should result in a decrease of the reduction current of PANI +350 mV by nanomolar concentrations of TAM [15]. They postulated that TAM should be reduced by oxygen in the presence of HRP—a reaction which is not possible.

A real alternative for decreasing the electrode potential for the indication of TAM has been presented by Gilardi's group: TAM is converted by bioelectrocatalysis using flavin monooxygenase into the respective TAM N-oxide and the oxygen consumption is indicated at −600 mV [16].

## 4. Conclusions and Outlook

Whenever the indication of the target binding by the redox marker ferricyanide is widely used to characterise MIPs, it is an “indirect” method. The decrease of the permeation may not only reflect “shrinking of pathways” by rebinding of the target. Also non-specific effects on the MIP structure by sample constituents can cause a decrease of the current. The direct electrochemical indication of the target is a more straigthforward strategy. We demonstrated this principle for TAM by indicating the anodic oxidation at the MIP–covered electrode. However, the oxidation of TAM brought about a fouling of the electrode surface. In order to prevent this adverse effect another electrode reaction has to be applied. Recently we demonstrated for the drug aminopyrine that enzymatic conversion of the target before the recognition by the MIP eliminates both fouling of the electrode surface and interferences by electroactive substances [6].

In preliminary experiments we found that pre-treatment of TAM with hydrogen peroxide in the presence of HRP generated an oxidation product which is reducible at 0 mV. At this potential the fouling of the electrode by the formation of a polymer film is circumvented. In the present stage of development the enzymatic reaction has to be performed in solution because the harsh regeneration of the MIP is not compatible with the stability of the enzyme.
